# Supplementary material for: Profiling of Essential Oils from the Leaves of Pistacia lentiscus Collected in the Algerian Region of Tizi-Ouzou: Evidence of Chemical Variations Associated with Climatic Contrasts between Littoral and Mountain Samples
Source: Molecules. 2022 Jun 28;27(13):4148. doi: 10.3390/molecules27134148 (PMC9268259; doi:10.3390/molecules27134148)
Supplement: Supplementary file 1 [file molecules-27-04148-s001.zip › molecules-1711866-supplementary.pdf]

Table S1. Weather data collected between 1999 and 2019 in Ait Irane, Algeria (https://fr.climate-data.org/afrique/algerie/tizi-ouzou/ait-irane-693594/).

	January	February	March	April	May	June	July	August	September	October	November	December
Mean temperature (°C)	5.2	5.3	8.5	11.6	15.6	20.8	24.8	24.5	20	15.9	9.4	6.2
Mean minimum temperature (°C)	0.8	0.5	3.1	5.8	9.4	13.9	17.5	17.8	14.5	10.7	5.2	2.1
Mean maximum temperature (°C)	10.4	10.7	14.3	17.5	21.6	27.4	31.9	31.6	26.3	21.9	14.5	11.2
Precipitation (mm)	88	78	84	86	74	21	8	19	53	60	81	82
Humidity (%)	79	77	73	70	67	55	45	47	59	64	75	79
Rainy days (jree)	9	8	8	8	7	3	2	4	7	7	9	8
Hours of sunshine (h)	5.7	6.4	7.6	9	10.3	12.3	12.6	11.7	9.9	8.4	6.4	5.7

Table S2. Weather data collected between 1999 and 2019 at Tigzirt, Algeria (https://fr.climate-data.org/afrique/algerie/tizi-ouzou/tigzirt-321206/).

	January	February	March	April	May	June	July	August	September	October	November	December
Mean temperature (°C)	10.2	10	12.1	14.2	17.1	21.1	24.4	25	22.3	19.3	14.1	11.3
Mean minimum temperature (°C)	7.7	7.4	9.3	11.3	14.1	17.9	21.2	22	19.6	16.5	11.7	8.9
Mean maximum temperature (°C)	12.8	12.7	14.8	16.8	19.7	23.9	27.3	27.9	25	22.2	16.8	13.9
Precipitation (mm)	142	107	86	68	51	9	2	6	36	73	140	146
Humidity(%)	75	74	75	77	77	72	69	69	71	71	73	74
Rainy days (jree)	10	8	7	6	4	1	0	1	4	6	10	10
Hours of sunshine (h)	7	7.8	9	10.2	11.1	12.2	12.3	11.3	10.1	9.1	7.6	7
